# Photothermal Effects of High-Energy Photobiomodulation Therapies: An In Vitro Investigation

**DOI:** 10.3390/biomedicines11061634

**Published:** 2023-06-04

**Authors:** Mark Cronshaw, Steven Parker, Martin Grootveld, Edward Lynch

**Affiliations:** Leicester School of Pharmacy, De Montfort University, Leicester LE1 9BH, UK

**Keywords:** dosimetry, parameters, photobiomodulation, thermography

## Abstract

The purpose of this study was to investigate photothermal aspects of photobiomodulation therapies (PBMT) in vitro to assist in the development of safe clinical parameters with respect to higher-power devices with large surface applicators. Laser wavelengths in the range of 650 nm–1064 nm were investigated using a thermal camera. Thermographic measures of surface and sub-surface temperature variations of similar lean porcine muscle tissue samples were recorded for a series of calibrated experiments. A thermal comparison was then made between Flat-top and Gaussian beam spatial distribution devices. Outcome data were subjected to statistical analysis using an ANOVA model. Results acquired at similar parameters of irradiance indicated that the application of the 980 nm wavelength was associated with the highest rise in temperature, which decreased with other wavelengths in the order 980 > 1064 ≈ 650 >>> 810 nm (*p* < 5 × 10^−20^). All wavelengths assessed were associated with a significant temperature increase, and with the exception of 810 nm, all exceeded the threshold of a 6 °C rise within the prescribed parameter limits. Optical scanning by movement of the applied source over a relevant area was found to offer effective mitigation of these temperature increases. An extended discussion is presented, analysing the clinical significance of the study outcomes. Recommendations are made within the limits of this in vitro study in order to assist future clinical investigations.

## 1. Introduction

Photobiomodulation therapy (PBMT) is the targeted application of light for therapeutic purposes at a level below that associated with damage to structural proteins. The capacity of laser light to modulate biological systems was first identified in animal studies conducted by Dr Endre Mester, and from 1971 onwards, human studies for a variety of musculoskeletal inflammatory disorders were conducted as an adjunct to promote good-quality healing. Consequently, there are now over 50 years of research involving tissue culture as well as animal and human clinical trials, and currently there is a very considerable published body of evidence in this area [1,2,3,4,5].

PBMT has attained a high level of scientific acceptance for the safe and effective prophylaxis and management of some clinical conditions, most notably in oncology in relation to oral mucositis [6,7,8,9,10,11], as well as a moderate body of evidence regarding many other uses, including the relief of pain and inflammation, as well as a variety of orthodontic and orthopaedic conditions [12,13,14,15,16]. However, the clinical protocols associated with consistent satisfactory outcomes in pain management, as well as in other potentially useful clinical applications, have not been fully defined [4,17,18].

During therapeutic laser application at the tissue surface, a high degree of energy loss at the underlying target occurs because of reflection, optical remittance, absorption, and optical scatter [19,20,21]. In recognition of the difficulties associated with optical transport to sub-surface target tissues, high-energy systems for the therapeutic delivery of an effective dose have become available. To facilitate the exposure of larger surface areas and volumes of tissue, large surface applicators are applied in conjunction with correspondingly higher output powers than was possible with the applicators previously used with single- or multiple-small-point applications. The use of larger surface optical spot sizes has been identified to be a successful strategy for improving the anticipated clinical outcome(s) [18,22,23,24,25]. However, these laser devices and the associated optics usually exhibit an inherent Gaussian distribution of power output, and the majority of the applied energy is located within the central third of the beam. This can result in difficulties delivering an even and accurate dosimetry. Furthermore, since applicators with larger surface areas require a higher output power to achieve the same average irradiance as a smaller applicator, there is the potential of exceeding the desired dose limits in the central zone, while underexposing the area in the peripheral third of the beam. Moreover, inadvertently high central-core-area irradiance may result in adverse effects culminating in phototoxicity, and could give rise to photothermally induced damage to the superficial tissues. 

In order to achieve a “safe” therapeutic level, current suggested guidelines recommend that the irradiance for photobiomodulation therapies remains below 0.75 W/cm^2^ when using near infrared sources (NIR), with a lower threshold limit of 0.3 W/cm^2^ for 600–700 nm sources, and 0.1 W/cm^2^ for 400–500 nm sources [26,27,28,29]. Many of the earlier-generation PBM therapy devices employ a small optic probe, typically with a low power output of between 0.005 and 0.15 W. Given a small superficial target area, and a probe typically 2–4 mm in diameter, red- to NIR-wavelength lower-power devices do not give rise to a rapid inadvertent temperature rise [30,31,32]. However, as confirmed by several recent systematic reviews and analyses, the usage of low-power-output small-area probes is a less successful strategy than that involving a larger area higher output power device in order to achieve a predictable clinical outcome in the management of some superficial, and most particularly sub-surface, conditions [4,18]. 

In the authors’ opinion, the reasons for the mixed research outcomes reported in the literature when small optic probes are employed is related to the issue of achieving radiant exposure (J/cm^2^) adequate for targeting tissues over larger areas, particularly in the case of some sub-surface conditions, where the treatment area may extend to several cm^2^ or more. Attenuation of the applied beam, in view of optical scatter in the red to NIR wavelength regions, reduces photon delivery at a depth of 5–10 mm to approximately 5–10% of the dose applied to the surface [33,34,35,36,37,38,39,40]. To compensate for this energy loss, it is therefore necessary to increase the dose delivered to the surface by at least 10-fold in order to achieve the desired value at a depth of 10 mm [4,38,39,40]. 

The benefits of using a larger surface beam include the facilitation of the exposure of larger areas in a shorter time. Additionally, there is a greater volume of delivered photons within the tissues which may assist optical transport to deeper tissue depths. By necessity, these devices require a higher overall power output in order to achieve the same average level of irradiance over a larger area of exposure as a smaller probe device. 

Most therapeutic laser systems use a base transmission emission mode (TEM_00_) with a typical beam divergence angle of around 16°. Aside from the uneven energy distribution across the beam, beam divergence can complicate dosimetry if a spacer is not used to assist the clinician to deliver the dose to a pre-determined surface spot size. However, the exception is the case of optically corrected devices such as a Flat-top hand piece, where, although it is the same source TEM_00_, the use of internal optical corrective devices such as prisms and lenses can cause the beam spectral profile to be ‘flattened out’, and the beam is collimated to a uniform spot size even at some distance to the target [41,42,43,44,45,46,47].

In the case of a typical TEM_00_ Gaussian beam, the spectral beam power profile peaks in the centre, with approximately 68% of the total power being in the central third of the area exposed. With a small probe and a correspondingly lower-output power device, this has not clinically been found to represent a major issue in the management of superficial conditions such as oral mucositis. However, this requires many more points of application, and substantially more time and operator skill to treat an area such as the oral cavity [7,48,49].

Optical transport in the red to NIR wavelengths is primarily forwards, with significant energy losses to depth, consequent to scatter and remission, a process resulting in a concentration of photons within the immediate boundary of the epidermis to the dermis [34,35,36,37,40]. Optical scatter predominates over absorption, permitting the delivery of photons to sub-surface tissues. There are, however, differences in the absorption and scattering coefficients of the wavelengths in the visible-red to NIR spectral range in relation to the components of the irradiated tissues such as water, fat, melanin and blood [21]. Absorption can result in a photothermal response, and the effects of a temperature rise produce biological effects ranging from some gentle stimulation of physiological processes to heat-induced cellular inhibition associated with a protective hormetic response up to the permanent denaturation of structural proteins, and overt photothermally induced damage at sustained temperature elevations above 45 °C [50,51,52,53,54,55]. The choice of which wavelength is best to apply for therapeutic purposes has been the subject of much controversy, and this is an area subject to continued research and evaluation [5,56,57,58,59,60].

The aim of this in vitro study was to investigate the surface and sub-surface thermal effects of wavelength, spectral beam profile, varying sizes of surface applicators and irradiance on the sample tissue of various laser diode sources used for PBMT. The photothermal aspects of laser–tissue interaction have been the subject of a considerable number of prior studies. However, there are no substantive in vitro studies quantifying surface and sub-surface thermal aspects of high-intensity laser PBMT devices while applying the variables of wavelength, spectral beam profile, beam area and technique (i.e., static vs. scanning methods). The therapeutic implications of the temperature increase associated with PBMT may be clinically significant with regards to achieving the desired outcome in a safe and consistent manner. Within the limitations of the results from an in vitro investigation, the potential relevance of the work conducted is discussed in detail.

## 2. Materials and Methods

**Figure 1:**
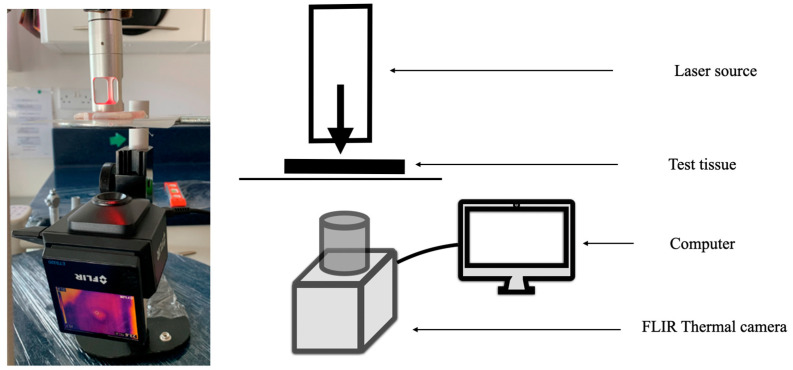
Test setup: Laser source irradiates test tissues. Thermal measurements (FLIR ETS-320 camera) were recorded as video (FLIR Thermal Studio Pro, Teledyne FLIR, Wilsonville, OR, USA). Transillumination: thermal measurements were taken on five sets of samples (single use only). Three different-sized applicators were employed, with areas corresponding to 1.0 cm^2^, 4.9 cm^2^ (shown) and 12.5 cm^2^. Images of the setup for surface and scanning modes are available in the Supplementary Materials Figures S1 and S2.

A test rig was constructed to permit standardised reproducible outcome measurements of multiple sets of tissue samples under a wide range of conditions, as described below (Figure 1).

Surface and sub-surface temperature measures of standard-sized 3, 5, or 20 mm thick lean muscle porcine tissue samples, sourced from a local retail outlet, were recorded using a calibrated video thermal camera system (FLIR ETS-320; Teledyne FLIR, Wilsonville, OR, USA), and a minimum of 5 sets of individual sample reading measurements were recorded using multiple same-thickness tissue samples. Single frames from the video were subject to analysis to identify the peak temperature in the zone of the tissue irradiated at the designated time periods (see Supplementary Figure S3). The thickness of each tissue sample was verified on the basis of three measurements using an Iwanson calliper gauge under 4× magnification to a tolerance of +/−0.5 mm. The wavelengths adopted were 650 nm, 810 nm, 980 nm and 1064 nm using laser diode sources as listed (Table 1). All laser source power outputs were calibrated to match the required output power using a power meter (Thor PM160; Thorlabs Inc., Bergkirchen, Germany). Average surface irradiance parameters ranged from 0.25–2.00 W/cm^2^ for the comparison of surface temperature between wavelengths, as well as the use of a Gaussian as opposed to a Flat-top optical beam profile device. All laser devices employed shared the inherent beam divergence properties of all TEM_00_ laser sources, and no polarising filters were applied. A power output range of 0.25–6.25 Watts, continuous wave (CW) was adopted for the measures of possible temperature increase variance using differing sizes of optical surface spot. Details of the power output ranges available from the devices and the parameter ranges employed can be observed in Table 1, with details of the successive categories of measurements taken being available in Table 2.

In order to assess sub-surface temperature changes, the 810 and 980 nm wavelengths applied were subjected to a comparative study. Firstly, different levels of irradiance in the 0.25–1.0 W/cm^2^ range were determined at the surface using a laser source with optics that delivered a Gaussian distribution of power output.

Secondly, the same laser wavelength with and without a proprietary optical attachment (Dr. Smile/Lambda It.), designed to correct the Gaussian beam to a top hat (Flat-top) beam profile, was assessed at 1.00 W/cm^2^. Both peripheral applicators provided a measured collimated beam area of 1.0 cm^2^.

Subsequently, a comparative thermographic assessment of the 980 nm diode laser was performed at an average irradiance of 0.25 W/cm^2^ with 3 different applicators with spot sizes of 1.0 cm^2^, 4.9 cm^2^ and 12.5 cm^2^. A further set of measurements was taken using the 810 nm diode laser and 12.5 cm^2^ spot size applicator at an average irradiance of 0.5 W/cm^2^.

In a later series, a scanning method was adopted in which a predefined area of 3.0 cm^2^ was exposed to a moving 1.0 cm^2^ collimated beam, and as with the static group, surface and sub-surface temperatures were recorded for fixed intervals of 240 s. Comparisons were also made between a Gaussian spatial distribution and a Flat-top beam for surface temperature changes at a standardised power output of 1.00 W/cm ^2^. As a notional ‘safe’ limit in temperature rise, we employed a threshold of an increase in 6 °C from the standard starting temperature of room temperature at 20 °C.

For statistical analysis, we applied an ANOVA model to determine the statistical significance of any differences or effects found. Post hoc analyses of differences between individual factor classification mean values for this model were performed using a Bonferroni testing system, along with plots of means with associated 95% confidence intervals. *XLSTAT2021* software (Addinsoft, Paris, France) was utilised for this purpose.

In cases where evaluations of the normality of data (test) and/or the homoscesdasticity (intra-sample variance equivalence) of sampling groups were found to be statistically significant, and hence deviate from these requirements, datasets were primarily log_10_-transformed prior to analysis. However, if this transformation was not found to be effective at normalising and/or ensuring variance homogeneity, then the robust Welch and Brown–Forsyth F ratio tests were applied to overcome these issues.

The reproducibilities (precision) of treatment-induced temperature rises observed were determined by computation of coefficients of variation (CVs) ‘between replicates’ for each experimental system evaluated (representing the ‘between-replicate’ CV values divided by their corresponding group replicate means). In all, a total of 36 sets of these experiments were conducted, and these included from *n* = 5 up to as many as 17 replicates. These CV values were found to range from 2.90 to 26.90%, with a mean ± SD value of 10.34 ± 6.81% (median value 7.55%). Of these, 11 were ≤5%, 10 were 5–10%, 11 were 10–20%, and only 4 were 20–30%. The 95% confidence intervals for the mean CV value were 8.04–12.62%. Hence, for this class of study involving prior- and post-treatment temperature readings, ‘between-replicate’ agreements in the majority of experiments conducted were entirely satisfactory.

## 3. Results

**Figure 2:**
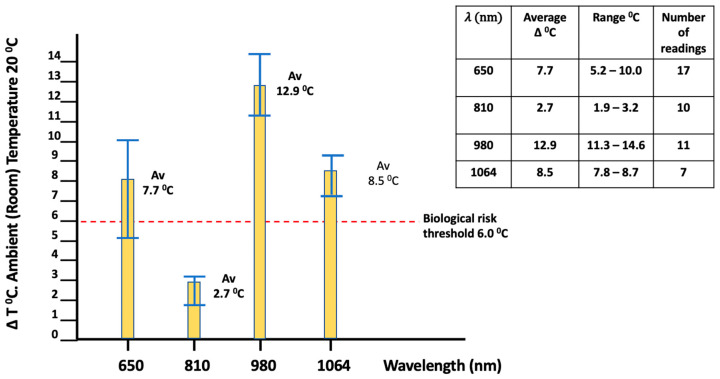
In vitro porcine muscle comparison of surface ΔT values as a function of wavelength (*λ*). Average power: 2.0 W/cm^2^ (continuous wave). Surface temperature increases exceeded the 6.0 °C threshold limit for all wavelengths except 810 nm.

The outcomes demonstrated a significant increase in surface temperature consequent to radiant exposure. Indeed, the temperature differential outcome ‘between wavelengths’ indicated that the temperature rose to above the 6 °C increase threshold value for all the wavelengths assessed, with the exception of the 810 nm wavelength at the 60 s mark with identical parameter settings for spot size and output power (Figure 2). Statistical analysis of the temperature elevation dataset acquired demonstrated that these differences were indeed extremely significant, with *p* = 3.29 × 10^−20^ and 1.43 × 10^−20^ for robust Welch and Brown–Forsyth F ratio tests, respectively. Moreover, post hoc analysis of these data revealed that the order of statistical significance was 980 > 1064 ≈ 650 >>> 810 nm, i.e., the temperature increase observed with the application of the 980 nm wavelength was significantly greater than with the application of any other wavelength; no significant differences were found between the mean temperature increases with application of the 650 nm and 1064 nm wavelengths, although the increases found for these were both significantly greater than that observed at 810 nm. Hence, the highest surface temperature values observed ‘between wavelengths’ throughout the 650–1064 nm range were those with the 980 nm sources.

**Figure 3:**
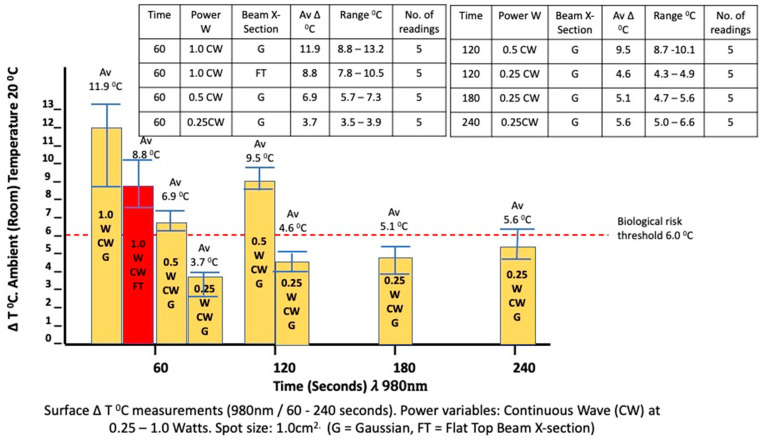
In vitro porcine muscle surface ΔT °C measurements (980 nm/60–240 s). Power variables: Continuous wave (CW) at 0.25–1.0 Watts. Spot size: 1.0 cm^2^. (G = Gaussian, FT = Flat-top beam X-section). The inset table represents details of the parameters applied for each result obtained.

It is, however, noteworthy that at 0.25 W and at extended exposure times of up to 240 s of continuous wave exposure with a static beam of 980 nm laser and a surface optical spot size of 1.00 cm^2^, the suggested risk threshold value of 6 °C was not exceeded (mean increase 3.72 °C, *p* < 10^−4^). However, at higher levels of irradiance of 0.50 and 1.00 W/cm^2^, the outcome was in excess of the threshold value of 6 °C (Figure 3).

An assessment of the change in surface temperature when employing the Flat-top device with the beam spectral profile compared to what was observed with the Gaussian beam is also presented in Figure 3. ⍙T measurements of the 980 nm source at 1.0 W/cm^2^ using the Flat-top device indicated a thermal increase at the surface to beyond the risk threshold, reaching Δ8.8 °C after 60 s. In contrast, the Gaussian device resulted in significantly higher surface temperatures than the Flat-top applicator, with the mean value for the former being approximately 25% higher than that for the latter (*p* = 0.022).

**Figure 4:**
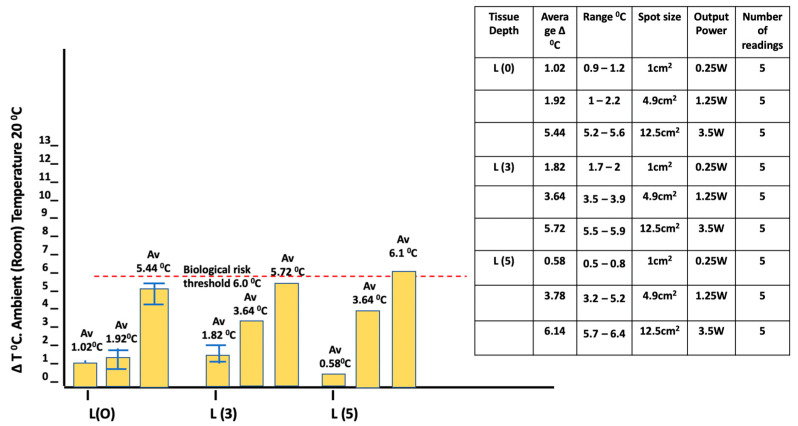
In vitro porcine muscle ΔT °C measurements (980 nm/30 s/static). Power: 0.25 W–3.5 W. Continuous wave. Spot size: 1.0–12.5 cm^2^. Gaussian beam. (Readings: L (0)—surface; L (3)—3 mm depth; L (5)—5 mm depth). Larger area applicators were associated with a progressive increase in surface and sub-surface ΔT value.

A comparison of thermal changes when using spot applicators of varying sizes set at the same average irradiance of 0.25 W/cm^2^ and applying a wavelength of 980 nm reveals a significant difference between the applicators observed at the surface, as well as at depths of both 3 and 5 mm (*p* = 5.03 × 10^−7^, 1.18 × 10^−7^, and 4.52 × 10^−3^, respectively). The largest applicator, with an area of 12.5 cm^2^, generated a 5.4 °C increase in temperature after 30 s at the surface, as well as a significant temperature increase at depths of 3 and 5 mm in the tissues monitored (Figure 4).

**Figure 5:**
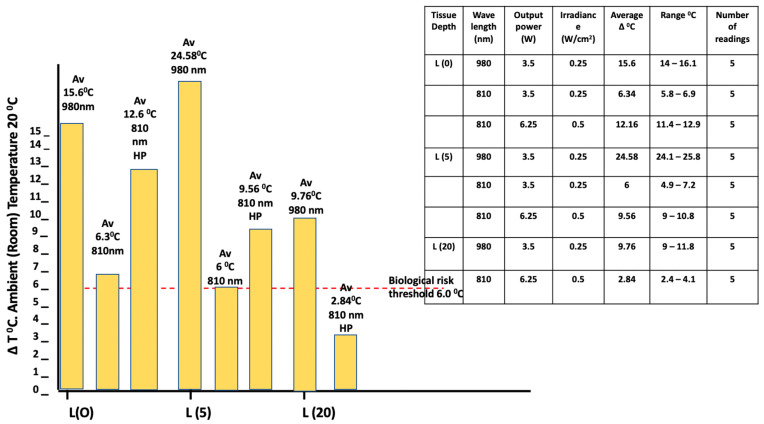
In vitro porcine muscle ΔT °C measurements (980 & 810 nm/180 s/static). Power: 3.5 W and 6.25 W (810 nm only). Continuous wave. Irradiance 0.25 W/cm^2^ and 0.5 W/cm^2^ (810 nm only). Spot size: 12.5 cm^2^. Gaussian beam. (Readings: L (0)—surface; L (5)—5 mm depth; L (20)—20 mm depth).

A comparison of the 980 and 810 nm diode laser sources using the same parameters of 0.25 W/cm^2^ and a large surface applicator of 12.5 cm^2^ for a period of 180 s provided evidence of marked increases in the surface and sub-surface temperatures for the 980 nm source compared to what as observed for the 810 nm one (*p* = 9.61 × 10^−9^ and 8.36 × 10^−10^ for the surface and 5 mm depth targets, respectively), with the latter remaining at or below the threshold of a 6 °C increase. However, at a higher irradiance of 0.5 W/cm^2^, with the 810 nm source, the tissue temperature increased at the surface by ca. 12 °C. A significant temperature increase was identified in thicker tissue samples up to a depth of 20 mm with both wavelengths (980 nm mean increase of 9.76 °C; 810 nm mean increase of 2.84 °C, *p* < 0.0001), although the parameters employed were different, since a higher irradiance of 0.5 W/cm^2^ was required to observe a significant effect at this thickness with the 810 nm device. (Figure 5).

**Figure 6:**
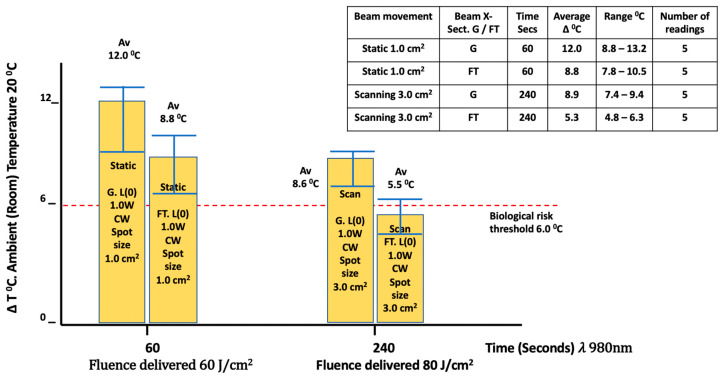
In vitro porcine muscle surface (L0) ΔT °C measurements (980 nm/60, 240 s). Power: continuous wave (CW) at 1.0 Watts. Flat-top beam vs. Gaussian. Spot size: 1.0 cm^2^ vs. 3.0 cm^2^. Comparison between static and scanning techniques.

The effects of optical scanning by moving the beam over a pre-determined area, as opposed to using a static beam, while obtaining an equivalent level of irradiance and radiant exposure was found to significantly facilitate the mitigation of excessive early temperature increases within the confines of the parameters employed (Figure 6). Mean ± SD values observed for these temperature increases in scanning modes with a collimated Flat-top beam were found to be 8.88 °C (static) at 60 s vs. 5.28 °C at 240 s (scanning). The corresponding outcomes for the collimated Gaussian beam were 12 °C at 60 s (static) vs. 8.8 °C at 240 s (scanning).

## 4. Discussion

The first law of photobiology requires that there is a cellular receiver for the transfer of energy from the incident photons to effect change. There are many potential subcellular sites for the absorption of light, including flavins, porphyrins, nuclear chromatin, cytochromes, opsins, cryptochromes and transiently reactive vanilloid potential membrane ion gates (TRPVs). Examples of endogenous chromophores include melanin, haemoglobin (oxy-haemoglobin, deoxyhaemoglobin and methaemoglobin), protein, peptide bonds, aromatic amino acids, urocanic acid and bilirubin. One common thread to these sites of energy transference are the presence of pigmented materials, which by virtue of their molecular density, are broad-band receivers of visible-to-NIR light. Should the resonant frequency of the incoming photons coincide with the valence band width of the target, this process can drive electrons into a higher valence band.

The potential energy of a photon is expressed by the term electron volt (eV), which is the amount of energy necessary to move an electron through a 1.0-volt electrical field. There is an inverse relationship between energy and wavelength, since shorter wavelengths have a higher cycle frequency and consequently a higher-energy electromagnetic field. As a consequence, the effects of a shorter-wavelength photon striking a molecular target are markedly different from those experienced at longer wavelengths. In red- to NIR-wavelength regions, there is insufficient energy to break the chemical bonds of many common tissue constituents, although this may be observed should a constituent molecule be subjected to two photons in simultaneous collision. It is recognised that although the red-to-NIR wavelengths are less than the threshold eV necessary to break chemical bonds, indirect photochemical-induced effects such as an increase in the production of ROS and NO^●^ are observed. The repeated and accumulated energy of a stream of photons can result in photothermal effects from the indirect kinetic effect consequent to an increase in elastic and inelastic molecular vibrations. Tissue consistency can change in response to temperature by virtue of an increase in molecular vibration (the Gruneissen parameter), as well as by vasodilatation or by vasoconstriction phenomena.

Tissues are, of course, not homogenous, and as the degree of anisotropy can change in response to temperature fluctuations, this affects the degree of optical scattering. With regard to the attenuation of optical penetration, this is a result of a combination of the degree of absorption and of scattering. Should there be a sustained increase in tissue temperature above 46 °C, this can result in protein coagulation which increases the coefficient of absorption (μ_a_) as well as increases in the coefficient of scattering (μ_s_). This impedes onwards optical transport and there can be a marked temperature increase within the tissues which, in view of the accumulation of applied energy, can produce higher sub-surface temperature rises than at the surface, as identified in Figure 4 and Figure 5. This effect may be more marked in vivo, as an overlying epidermis has been recognised to create boundary effects related to differences in the refractive indices of the epidermis as opposed to the dermis. As a consequence, there can be a higher photon density at the dermal–epidermal border, resulting in thermal conduction spreading laterally to as well as in parallel with the direction of the applied coherent laser photonic stream. Thermal transport takes place by radiation at the surface of the tissue samples, conduction within the tissue medium, and in vivo via convection by the arterial and venous blood supply. In response to a small elevation in tissue temperature, vasodilatation is observed in vivo, whereas vasoconstriction occurs at temperatures above 46 °C. The former is considered a protective response that carries away heat from the hyperthermic area. whereas the latter protects against the transport of excess heat to tissues beyond the hyperthermic region [19,20,21,22,49,61,62,63].

The outcomes of this study demonstrate that there can be a marked temperature increase associated with the applied parameters. The surface and sub-surface temperature increases achieved in vitro indicate that the recommended advisable physiological limits in vivo of a close to 6 °C rise above the normal body temperature of 37.5 °C could be exceeded. It is accepted that beyond this extra 6 °C level, there is the potential for permanent deformation of structural tissue proteins to occur, as well as perhaps other biomolecules, when subjected to protracted exposure to temperatures above 45 °C [50,51,52,53,54,55]. Indeed, many enzymes or other biomolecules are known to be significantly or substantially inactivated at this temperature. The higher the temperature increase beyond 45 °C, the shorter the duration for which the cell can tolerate the thermal stress, and the higher the corresponding risk of permanent harm to cells and tissues. Progressively steeper temperature increases above this threshold can culminate in cellular dysfunction, a process ultimately resulting in apoptosis. A further biochemical example involves the thermo-oxidation of membrane unsaturated fatty acids, particularly polyunsaturated fatty acids. As such, the temperature increases involved are of crucial importance. The potential for phototoxicity was investigated by Khan and Arany in a murine model, from which a pathway was characterised that involved a system of chaperone proteins referred to as Heat Stress Proteins (HSPs), which act in concert with a stress-induced protein called activated transforming factor 4 (ATF-4). The outcomes of higher-dose photoirradiation were identified to be temperature related, where even a mild increase in temperature quickly inactivated the ROS buffering enzymes catalase and glutathione reductase, and also resulted in strong activation of the heat stress protein (HSP) chaperone system. In response to photon-induced temperature elevations, an increase in ROS levels was noted, which, when in excess of the capacity of the cells’ normal antioxidant systems to reduce ROS activities, activated a sequence mediated by ATF-4, a process resulting in calcium ions being released from deposits in the endoplasmic reticulum. Elevated cytoplasmic calcium ions activated the opening of the mitochondrial transport pore, which, in parallel with the actions of the HSP cascade, effectively closed down mitochondrial activity and placed the cell into a temporary form of stasis [50].

**Figure 7:**
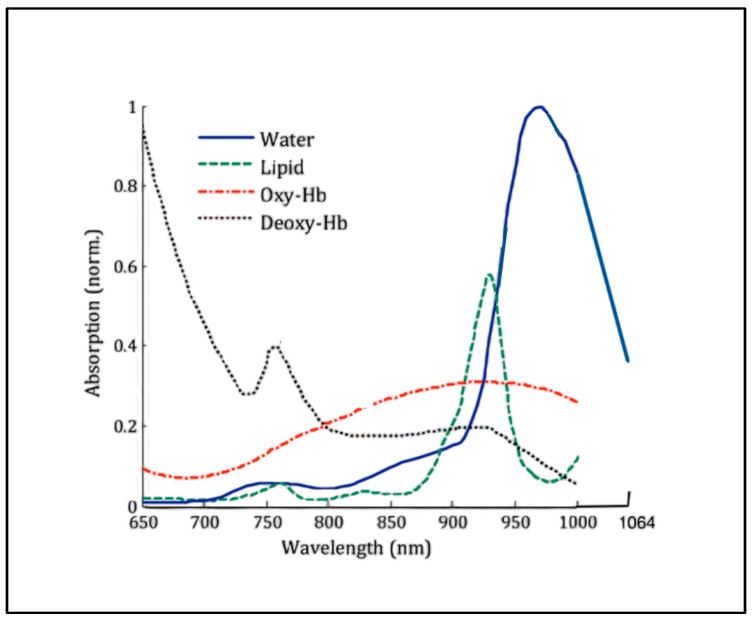
Normalised absorption spectra of water, lipid, and oxy- and deoxyhaemoglobin (Hb) in the near-infrared spectral window (adapted from Ruiz et al. [65]).

Surface thermography demonstrated that the greatest surface temperature increase in the unit time applied was that observed at 980 nm, in descending order as follows: 980 > 1064 ≈ 650 >>> 810 nm. With respect to sub-surface measures, application of the 980 nm wavelength with a larger applicator and the described parameters resulted in a marked increase in temperature at a depth of around 2 cm. In contrast, the 810 nm wavelength was associated with a slower rate of temperature increase, which may be beneficial for the application of high-intensity laser PBMT to sub-surface targets. The tissue absorption properties are directly related to the presence and concentration of light-absorbing molecules. The most important absorbing molecules relevant to the red-to-NIR range of wavelengths found in lean muscle tissues are water and haemoglobin [40,64,65] (Figure 7).

However, with visible-red to NIR optical sources, scattering is very much greater than absorption, with minimal values being centred at the 800 nm level, and this wavelength is recognised as being close to optimal for depth penetration into tissues. In contrast to 810 nm, the 980 nm wavelength has a small absorption affinity for water, which by virtue of kinetics, creates an increase in molecular vibration, a process that results in heat generation [19,20,49,64]. Biological tissues have a high water content, with the average adult male body consisting of around 55% water by volume, while the female body has a slightly higher water content of around 60%. Since the rate of optical thermal generation is proportional to the prevalence of the absorbing medium, the absorption coefficient and the fluence rate, a 980 nm laser is capable of producing rapid localised heating in tissue [19,66].

Based on our in vitro investigations, in order to avoid excess heat accumulation, we therefore advocate caution in employing the 980 nm wavelength for extended therapies targeted at sub-surface tissues.

Our results demonstrate a significant temperature increase at a depth of 3–5 mm, with a marked difference in outcome depending on the size of the surface applicator. Although the average irradiance of the applied beam was maintained at 0.25 W/cm^2^, the largest temperature increase was associated with the larger applicator (Figure 4).

Larger surface optic applicators require a higher power output to achieve a comparable average irradiance to that of smaller optic spot devices. The Gaussian peak spectral beam profile effect in large applicators can be highly significant, as depicted in Figure 4 and Figure 5, where, although the same mean irradiance was applied, there is a highly significant progressive difference in the observed temperature increase when using applicators with increasing spot size.

**Figure 8:**
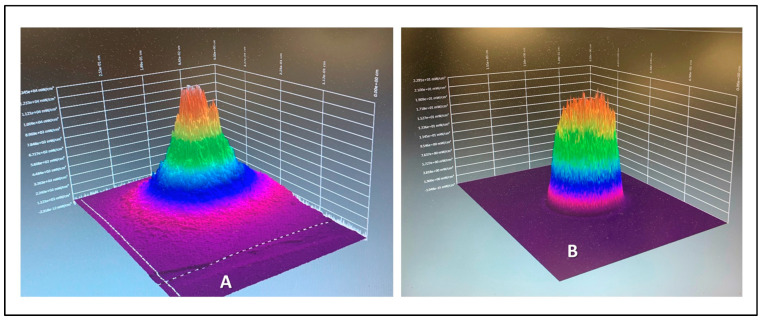
Comparison of a typical Gaussian beam distribution (**A**) and a Flat-top optically corrected beam profile (**B**). 3D render. Images solely for figurative demonstration and not to scale. Ophir beam profilometer, Beamgage v. 5.5 (Ophir Optronics LLC, Jerusalem, Israel).

The majority of optical spot size applicators are Gaussian in an optical beam spectral profile, and this complicates the calculations of safe delivery parameters, especially with larger surface spot sizes. Inherent to Gaussian devices is the fact that the power distribution typically generates a peak in the middle third of the beam (Figure 8).

**Figure 9:**
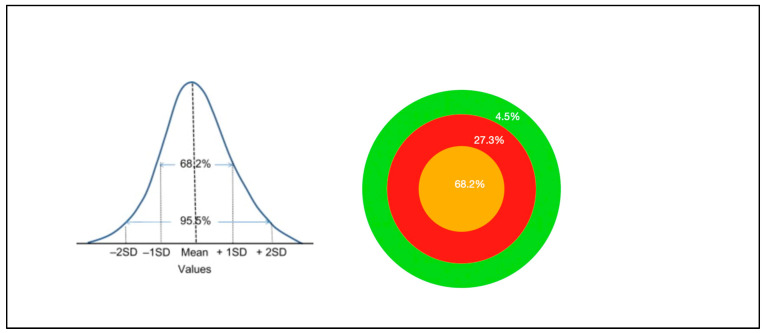
The optical power distribution of a typical Gaussian beam device predominantly lies within the centre of the beam.

Overall, since optical scatter in the red to NIR range is predominantly forwards, there is a tissue lensing effect amplified by the Gaussian spatial beam profile of the applied photonic delivery, with a higher concentration of photons within deeper tissues lying in the direct centre of the beam [2,4,12,42,48] (Figure 9).

The parameters we chose to use in the current study included settings of 2.00 W/cm^2^, which are far greater than the recommended upper limit proposed by Zein et al. of 0.75 W/cm^2^ for NIR PBM [28]. However, we regarded the higher irradiance setting as relevant for higher-output Gaussian devices, since with a greater irradiance surface area, the middle third of the beam can reach values in the region of 1–2 W/cm^2^ (Figure 9).

As illustrated, even at an average level of irradiance, far below the upper limits recommended for PBM, it is possible to significantly exceed the suggested NIR peak irradiance value of 0.75 W/cm^2^ for a significant area of tissue.

Kaub and Schmitz [40] employed a similar in vitro design to our own in order to investigate the optical attenuation of two 905 nm sources. Both were high-intensity devices emitting short-duration high-frequency pulses at a similar average power of 1–1.2 W. There were differences in parameters between the two sources, as the peak power of one (EMS) reached 300 W at 40 kHz in a 0.4% duty cycle, whereas the peak power with the other (K-laser) reached 20 W at 20 kHz in a 50% duty cycle. The outcome was found to be around 90% attenuation at a tissue depth of 7 mm for both sources, with transmittance between 0.3–0.4% at a depth of 20 mm. Given the high degree of attenuation, it would be expected that any temperature increase within the tissues would be primarily identified within the first 10 mm of tissue. Our outcome is in accordance with the concentration of photons, which produce some photothermal effects in the more superficial layers of tissues. However, we also found a significant temperature rise of 2.84 °C at a depth of 20 mm when applying a large surface applicator with an area of 12.5 cm^2^, with an 810 nm source applied at an average irradiance of 0.5 W/cm^2^. Moreover, a temperature rise of 9.76 °C was observed when applying the 980 nm source at an average irradiance of 0.25 W/cm^2^ (Figure 5). Given an appreciation of the significance of the Gaussian power distribution, we consider that this result is most likely attributable to the core area irradiance being in the vicinity of 2–4 W/cm ^2^ when applying the 810 nm wavelength, and 1–2 W/cm^2^ for the 980 nm source.

**Figure 10:**
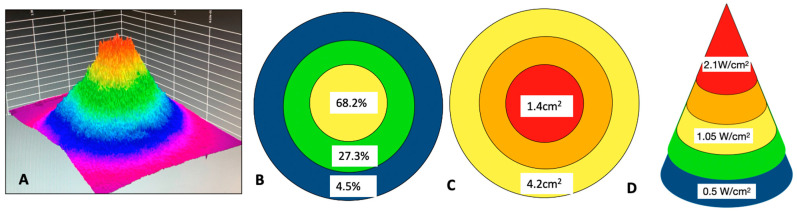
(**A**) Gaussian beam profilometer 3D render of a 4-cm-diameter applicator with a surface beam area of 12.5 cm^2^. (**B**) Approximately 68% of the power is concentrated in the middle third of the beam, with around 27% in the middle third and less than 5% in the outer third. (**C**) In the middle third of the beam, the area is approximately 4.2 cm^2^ and the power further progresses to a peak in the middle third of this central area. (**D**) At a power output of 6.25 W, the average irradiance across the entire area of 12.5 cm^2^ is 0.5 W/cm^2^. However, as indicated above, the average irradiance in the central area is 1.05 W/cm^2^, with a further peak within the innermost central zone in excess of 2.1 W/cm^2^.

Based on our study, we recommend, for the purposes of scientific reproducibility, that a calculation be performed of the irradiance of the middle third of the beam, with a further calculation for very large applicators with a central core zone of 1 cm^2^, with the objective of circumventing the limit of 0.75 W/cm^2^ using NIR wavelengths, 0.3 W/cm^2^ for 600–700 nm wavelengths, and 0.1 W/cm^2^ for 400–500 nm wavelengths being exceeded. In clinical practice, we propose that the clinician should be made aware that the central third of the beam will reach an average irradiance of double the average irradiance of the total beam area, and furthermore, that in the case of larger applicators of several cm^2^ and above, the innermost area will reach an irradiance of more than four times the average irradiance (Figure 10A–D).

The outcome described here regarding use of a Flat-top device shows that in comparison to a Gaussian beam, there was a reduction in the surface temperature rise both in the static and scanning modes. At 1 W/cm^2^, the surface temperature in combination with the 980 nm wavelength resulted in a 8.8 °C temperature increase at 60 s in static mode. In scanning mode, a lower 5.5 °C rise was attained at 240 s, although the delivered fluence at 240 s of 80 J/cm^2^ was in excess of that of the static mode dose of 60 J/cm^2^. The corresponding values for the Gaussian beam; however, showed a 12 °C increase at 60 s in static mode, and an 8.6 °C temperature increase at 240 s in scanning mode. Clearly, our results indicate that there is merit in adopting an optical scanning mode using either device with some added benefits to the Flat-top peripheral applicator. However, we emphasise that the larger the applicator area, the greater the potential hazard for photothermal excess with a Gaussian-type device. Newer and larger Flat-top applicators with a diameter of 5 cm have been developed; pending further research, the merit of the Flat-top concept has yet to be proven clinically, although the authors view this as an interesting emerging technology [47].

It has previously been proposed that the Flat-top device may enhance optical delivery to depth as well as rationalise dosimetry [41,43,44,45,46]. However, at present the availability of this type of optically corrected device is restricted, and the currently available 1 cm^2^ spot size is of reduced utility in treating larger areas of tissues by comparison to the larger Gaussian applicators currently available.

Optical pathways of an incident coherent light source vary according to the angle of application, the refractive index of the medium, the wavelength applied and the physical structure and content of the exposed tissues. The directionality of the source may be polarised, and all diode laser sources have an inherent property of linear polarisation which, according to the angle of incidence, may increase the absorbance value of the incoming photonic energy without an appreciable associated incremental rise in tissue temperature compared to a non-polarised source. Although in some cellular and animal studies this effect has been noted to promote enhanced healing, the relative importance of this outcome is not known. Furthermore, it is also recognised that polarity is only maintained in the most superficial layers of the exposed tissues [67,68,69,70].

The limitations of our study are that we employed a single tissue type of lean porcine muscle without any overlying epidermal tissues or underlying adipose layers of tissue.

In view of differences in the scattering and absorption coefficients of keratin and adipose tissue, in vivo measurements of temperature increases associated with laser exposure may reasonably be expected to be at variance with our outcome. Our results here are intended purely to be indicative of the need for caution rather than prescriptive to any particular parameters for clinical patient applications.

There have been several recent animal studies that have investigated thermal effects associated at a single wavelength, as well those experienced with dual wavelength approaches to PBMT. Zielinska et al. investigated high-intensity laser therapy (HILT) in healthy pigmented and non-pigmented horses, with experiments performed on shaved fur measuring temperature rises, and vein diameters using a surface thermal camera and ultrasound [71]. A 5 cm^2^ probe in scanning mode was applied with an 808 nm laser source set at an average power of 4 W, with 2 kHz applied to an area of 26 cm^2^ over 203 s, a process delivering a total of 650 Joules. A significant increase was noted in the vein diameters in both groups, and also an average increase in surface skin temperature in the pigmented group only, by an average of 3.1 °C. In a more recent study, the same group assessed temperature increases and changes in pain on palpation in pigmented skin horses with back pain [72]. In this latter study, a simultaneous delivery of 808 and 980 nm wavelengths were administered using a scanning method using the same 5 cm^2^ probe to a larger area of 100 cm^2^ using an average power of 5 W, with 100 Hz delivering an average radiant exposure of 20 J/cm^2^. An increase in surface temperature of 2.5 °C was observed, and a reduction of pain scores was also identified. The temperature increases at the skin surface were less than the outcome we report here at a similar irradiance of 1.0 W/cm^2^. However, there are several important differences between the animal studies and our own in vitro investigation. The total radiant exposure in the equine studies was in the range 20–25 J/cm^2^, whereas we applied 80 J/cm^2^. Furthermore, we used a continuous wave application, whereas in the equine studies, a gated pulse delivery was applied. There are several other important differences to be noted, since our experiments involved the exposure of non-pigmented, lean porcine muscle, whereas the live animal studies irradiated pigmented and non-pigmented skin. A further consideration is that we utilised a single wavelength of 980 nm at 1.0 W/cm^2^, whereas in the dual-wavelength equine study, the two wavelengths of 808 and 980 nm used were set at 0.50 W/cm^2^ for each wavelength in order to achieve the same irradiance value of 1.0 W/cm^2^. We identified here that the 980 nm source is a considerably ‘hotter’ wavelength than the 810 nm one (Table 2). Indeed, given the application of an overall higher irradiance in our study, and also the sole use of the 980 nm source to a smaller area, we do not view our results as anomalous.

Our assessments support the adoption of a scanning method with larger output devices, which within the limitations of our in vitro study appear to permit extended exposure to the tissues without an associated rapid temperature increase. 

It has been proposed that a highly localised low-level thermal increase may offer some benefits to cellular physiology. This could range from a moderate enhancement of enzymic processes through to the activation of protective hormetic responses, which may be associated with analgesia, along with a wide variety of associated responses, a process resulting in cellular, local tissue, regional and systemic effects Indeed, it is recognised by the dermatology community that a small incremental rise in temperature of 1–2 °C can enhance the quality of healing processes [73,74].

We observed in our study here that higher output power with large applicators was able to generate some heat in the surface and sub-surface tissues. Low levels of intracellular thermal increases within the range of 2–6 °C are not notably harmful, although they can trigger defensive protective cellular apparatus [75,76,77]. By the selective generation of small localised thermal inclines, it may be possible to inhibit axons and hence contribute to the desired effect of analgesia. The mechanisms underlying the analgesic effects of PBMT could conceivably include photon-induced axonal inhibition arising from a hormetic response to an increase in intra-cellular ROS generation, together with an associated temperature increase [17,54,78,79,80,81]. Previous studies have indicated that HILT increases blood flow in soft tissue, which creates a favourable environment for biological repair and regeneration and which also promotes analgesic effects [82,83].

Furthermore, we theorise here that photon transduction is a highly localised intracellular event associated with the red to near-infrared wavelengths, with transition metal ions incorporated into proteinaceous clusters as prosthetic groups, such as those which may be found in cytochromes, including units 3 and 4 of the Electron Transport Chain (ETC), membrane-bound cyclo-oxidase (COX-2), together with further porphyrin species and flavins. Furthermore, dark-pigmented materials such as nuclear chromatin are broad-band receivers of photonic energy, which can give rise to the generation of heat. There are also specific temperature and photon-responsive outer membrane ion gates known as transient receptor potential vanilloids (TRPVs). Moreover, other complex PBM pathway processes may possibly be associated with photomagnetic, photoelectric and photofluorescent phenomena [3,53,59,60].

Photonic exposure may generate intracellular micro-thermal inclines; however, any highly localised intracellular temperature increase generated by PBM is not readily amenable to measurement using standard thermocouples. Fortunately, newer tools for detecting microthermal inclines are now available, and this is an area we consider to be of interest for future research [84,85,86].

The current clinical evidence base has been found to support the requirement for a higher dosimetry to produce pain relief [17,54]. With the added appreciation of the possible significance of highly selective photothermal photon transduction processes, it may be possible to safely and predictably benefit from the judicious application of larger optical sources and a higher total energy delivery in order to generate some valuable clinical outcomes for the relief of pain.

Moreover, there is the evident need for continued research, and as an aid to this, it is hoped that this study, along with our previously published multiple systematic reviews and meta-analyses with extensive data extraction strategies, may prove to be of value, particularly with regard to safely achieving a potential analgesic effect.

## 5. Conclusions

An in vitro investigation of the photothermal effects of high-energy photobiomodulation therapies was undertaken. Based on the outcome of this study, the inherent issues associated with the Gaussian beam spectral profile with high output and large surface applicators indicates that the choice of wavelength and technique adopted is important for circumventing the overheating of surface and sub-surface tissues, particularly with a 980 nm source. With very large applicators, clinicians should be made aware that the irradiance of the central core of the beam may be 2- to 4-fold higher than the average irradiance. Additionally, for the purpose of achieving reproducibility of studies, a calculation of the innermost third peak irradiance is advisable. At the high powers associated with the larger applicators, the combination of a scanning technique with continuous monitoring of the surface temperature is advocated. The ability to safely deliver higher overall energies to treat larger volumes of tissues may open up many new therapeutic options for the application of PBMT, and the outcomes described here offer some substance to possible useful approaches to investigate in in vivo studies in the future.

Safe and consistently effective clinical parameters for many conditions possibly amenable to treatment with PBM have not as yet been defined, nor have they attained the required level of scientific scrutiny to permit acceptance of the validity of the methods described to the satisfaction of the broader evidence-based clinical community. The increased awareness of low-level 2–6 °C photothermal aspects to PBM in high-intensity laser applications may offer insight into some of the underlying mechanisms associated with this therapeutic approach in the safe administration of PBM dose to depth, as well as in relation to pain management.

## Figures and Tables

**Table 1:** Details of laser device wavelengths and parameter ranges employed in this study.

Laser Device	Wavelength *λ* (nm)	Emission Power Range (Watts)	Application Spot Area (cm^2^)
i-Lux XP (Mectronics Medicale, Grassobbio, Italy)	650/ 810/ 980/ 1064	0–2 0–10 0–10 0–10	1.0 1.0, 12.5 1.0, 4.9, 12.5 1.0
Wiser (Dr. Smile, Lambda Spa, Brendola, Italy)	980	0–8	1.0 (collimated Flat-top)

**Table 2:** Details of the successive categories of measurements, wavelengths, parameters employed and spectral beam profiles applied (Gaussian: G; Flat-top: F-T).

ΔT Values Surface/Sub-Surface (Depth: millimters	Wavelength *λ* (nm)	Application Spot Area	Power: Watts	Average Irradiance (W/cm^2^)	Time (seconds)	Beam Profile: Gaussian (G)/Flat-Top (F-T)	Results Figure
Surface	650/810/980/1064	1 cm^2^	1	1	60	G	2
Surface	980	1 cm^2^	0.25–1.0 1.0	0.25–1.0 1.0	60/120/180/240 60	G F-T	3
Surface & sub-surface 0/3/5	980	1 cm^2^ 1 cm^2^ 4.9 cm^2^ 12.5 cm^2^	0.25 0.25 1.25 6.25	0.25 0.25 0.25 0.25	30 ST	G FT G G	4
Surface & sub-surface 0/5/20	980 810 810	12.5 cm^2^ 12.5 cm^2^ 12.5 cm^2^	3.50 3.50 6.50	0.25 0.25 0.50	180 ST	G	5
Surface Static: ST Scanning: SC	980	1 cm^2^ ST 1 cm^2^ ST 3 cm^2^ SC 3 cm^2^ SC	1.0 1.0 1.0 1.0	1.0 1.0 1.0 1.0	60 60 240 240	G F-T G F-T	6

## Data Availability

The datasets used and analysed during this study are available from the corresponding author on reasonable request.

## Supplementary Materials

The following supporting information can be downloaded at: https://www.mdpi.com/article/10.3390/biomedicines11061634/s1, Figure S1. Surface temperatures measured using the FLIR ETS-320 (Teledyne, USA) thermal camera using a 1 cm^2^ collimated beam spot size. Five sets of standardised lean porcine muscle tissue samples each used one time only for the four wavelengths assessed. Focal length from tissue sample to thermal camera: 7 cm. All sources calibrated using Thor PM160 power meter (Thorlabs, Germany). Figure S2. A Gaussian beam was compared to a “Flat-top” optically corrected spectral beam profile, irradiance set at 1 W/cm^2^, grid area 3 cm^2^, five sets of samples and measurements for each device. Collimated static spot applied for 60 s then to a further set of samples with constant slow movement for 240 s. Surface temperatures measured before and after using the FLIR ETS-320 camera (not shown). Figure S3. Single frame image taken from a video of a 980 nm source with a 1 cm^2^ spot size at the selected time interval of 60 s. Peak surface temperature of 34 °C from an initial temperature of 20 °C.
