# Supplementary material for: Photothermal Effects of High-Energy Photobiomodulation Therapies: An In Vitro Investigation
Source: Biomedicines. 2023 Jun 4;11(6):1634. doi: 10.3390/biomedicines11061634 (PMC10295700; doi:10.3390/biomedicines11061634)
Supplement: Supplementary file 1 [file biomedicines-11-01634-s001.zip › biomedicines-2345156-supplementary.pdf]

Supplementary Materials:

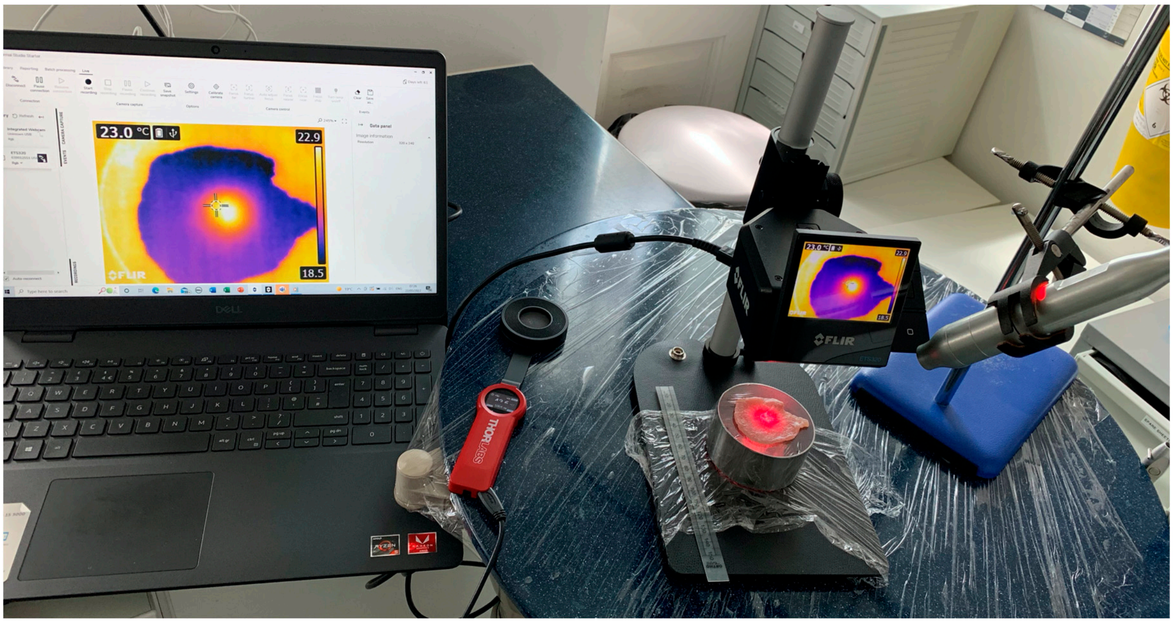

Figure S1. Surface temperatures measured using the FLIR ETS-320 (Teledyne, USA) thermal camera using a 1 cm^2 collimated beam spot size. Five sets of standardised lean porcine muscle tissue samples each used one time only for the four wavelengths assessed. Focal length from tissue sample to thermal camera: 7cm. All sources calibrated using Thor PM160 power meter (Thorlabs, Germany).

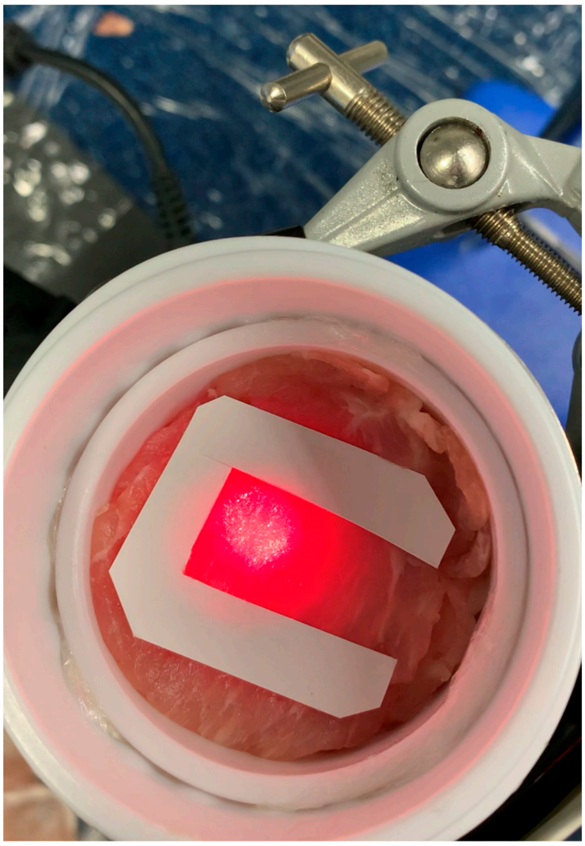

Figure S2. A Gaussian beam was compared to a "Flat-top" optically corrected spectral beam profile, irradiance set at 1W/cm^2, grid area 3cm^2, five sets of samples and measurements for each device. Collimated static spot applied for 60 seconds then to a further set of samples with constant slow movement for 240 seconds. Surface temperatures measured before and after using the FLIR ETS-320 camera (not shown).

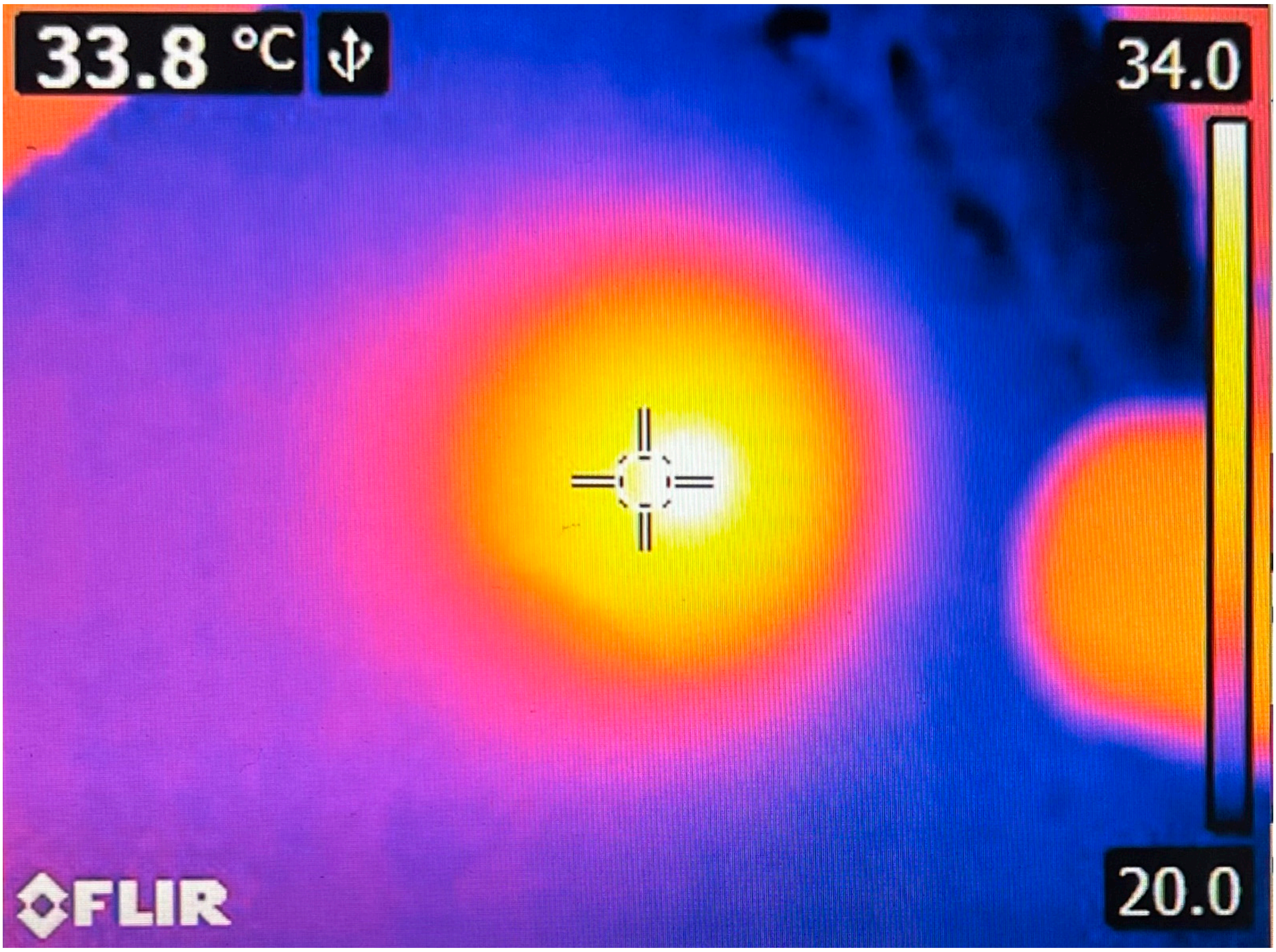

Figure S3. Single frame image taken from a video of a 980 nm source with a 1 cm^2 spot size at the selected time interval of 60 s. Peak surface temperature of 34°C from an initial temperature of 20 °C.
